# Rotheln

**Published:** 1881-07-20

**Authors:** A. B. Loving

**Affiliations:** Arkansas


### ROTHELN.

BY A. B. LOVING, M. D., OF ARKANSAS.

I see a communication in your last, from Dr. Knott, of Texas, in which he states that he has met with an eruptive disease, about which there seems to have been a diversity of opinion.

As I have had some cases the past spring, of a disease not unlike measles, which for a while led me off the track, I will give the history of them. Case No. 1. Mr. A. was taken with all the symptoms belonging to the forming stage of Rotheln. When first seen by me these were all well developed. There was the injected and watery eye, with continued sneezing; there was also sore throat. The eruption had made its appearance on the forehead, and in the pharynx. I diagnosed this meaales, but the course of the eruption was not that I had been in the habit of seeing in measles.

It spread too rapidly over the body, it did not cause that raised and thickened appearance of the skin that we see in rubeola, nor did it arrange itself in crescentic shaped spots; it did not remain on the patient long enough, and when it began to disappear it was off too soon, and without desqueamation.

The tongue was coated with a whitish brown coat, but this soon cleaned, leaving it looking like a piece of raw beef. I then began to think I had a case of scarlet fever, and made the following prescription:

| | | |
|---|---|---|
| ℞ | Fl. ext. belladonna | f. gtts. viii. |
| | Potassæ chl | ʒ ii. |
| | Aquæ | ℥ iii. |

M. Teaspoonful every two hours.

At my next visit twelve hours from that time tongue looked natural and the patient so much better that the idea of scarlet fever banished from my mind. This case was down seven or eight days, but the eruption all gone at the end of the fourth day.

Case No. 2. Mr. T's case run the same course as did Mr. A., but did not have the forming symptoms of measles so well developed. The tongue was the same, and yielded as promptly to the belladonna and potash prescription as the first case.

This young man claimed that he had the measles when a child, "Big French measles," and knew he could not have it again, but gave it up when he saw himself in a glass. All the other cases were in a mild form, and attacked those who had had measles as well as those who had not, and seemed to attack by preference, adults rather than children. It did not spread by contagion, as those who did not come in contact had the disease. My opinion after the first two cases was, that I did not have rubeola nor scarlet fever, but rotheln.

You can see from this how easy it would be for one to make a mistake in favor of either disease, rubeola or scarlet fever.

